# Endovascular treatment of traumatic dissection of the thoracic aorta – series of 16 cases

**DOI:** 10.1590/1677-5449.200074

**Published:** 2020-11-16

**Authors:** Lucas Mansano Sarquis, Wilson Michaelis, Antonio Lacerda Santos, Cristiano Silva Pinto, Rogerio Akira Yokoyama, Erick Fernando Seguro, Antonio Luiz da Costa Martins, Vinicius Belas do Vale

**Affiliations:** 1 Hospital do Trabalhador – HT, Serviço de Cirurgia Vascular, Curitiba, PR, Brasil.; 2 Hospital Universitário Evangélico Mackenzie – HUEM, Serviço de Cirurgia Vascular, Curitiba, PR, Brasil.; 3 Universidade Federal do Paraná – UFPR, Faculdade de Medicina, Curitiba, PR, Brasil.

**Keywords:** thoracic aorta, wounds, aortic diseases, endovascular procedures

## Abstract

**Background:**

Aortic injuries caused by blunt chest traumas have high pre-hospital and emergency mortality. The endovascular approach is one option for treatment of these injuries, but many outcomes related to this approach remain unknown.

**Objectives:**

The aim of this study is to describe a specialist trauma center’s experience with endovascular treatment of cases like these.

**Methods:**

This is a descriptive study based on review of the electronic medical records of patients who had suffered from blunt thoracic aorta trauma and were seen at a hospital specializing in trauma cases in the city of Curitiba (Paraná, Brazil).

**Results:**

Sixteen patients were included in the study. All patients were traffic accident victims and 75% of the accidents were the result of vehicle collisions. Aortic lesions ranged from grade I to IV and the majority had grade II lesions (50%). All patients underwent endovascular treatment with endografts, an average of 71 hours after the trauma. Two patients died, both from causes unrelated to their aortic injuries. During follow-up, only two patients presented complications (endoleak and progression of the dissection).

**Conclusions:**

The endovascular method is a viable alternative for treatment of blunt trauma thoracic aortic injuries. Randomized and controlled studies are needed to provide evidence to support indication of this method to treat this type of injury.

## INTRODUCTION

Blunt thoracic aortic trauma is the second most common cause of death of trauma patients, with a pre-hospital mortality rate of 80%, second only to head trauma.^1^^,^^2^ The main risk factor for thoracic aorta injury in blunt trauma cases is sudden deceleration, and the most prevalent trauma mechanisms are automobile collisions (70%), motorcycle accidents, falls from height, and being run over.^3^ This type of injury is involved in one third of deaths caused by automobile collisions.^4^

The majority of blunt thoracic aorta trauma victims are young adults of working age, presenting with involvement of multiple systems,^1^ with injuries primarily concentrated in the head, abdominal area, and/or lower limbs.^5^ Conventional surgical treatment can involve addition risk for multiple trauma victims and, in view of this, endovascular treatment offers a promising alternative approach for treating blunt trauma injuries involving the thoracic aorta.^1^

Considerable advances have been achieved over the last 20 years in the treatment of patients who remain alive long enough to be treated in hospital. This has occurred thanks to development of pre-hospital systems, training of emergency teams, expansion of diagnostic resources, such as computed tomography (CT), and endovascular methods.^4^^,^^5^ Endovascular treatment has become the first-choice method for hemodynamically stable patients with thoracic aorta injuries caused by blunt trauma and is associated with lower mortality rates when compared with conventional surgical treatment.^6^

Injuries to the aorta caused by blunt chest traumas range from lacerations involving the tunica intima of the vessel to complete rupture of the vessel wall,^7^^,^^8^ and the portion most often injured is at the ligamentum arteriosum, a section immediately distal of the left subclavian artery, which is a region of transition between the aortic arch (relatively mobile) and the descending aorta (more fixed).^2^

The most widely employed classification for traumatic thoracic aorta injuries was proposed by Khoynezhad et al.^7^ and adopted by the Society for Vascular Surgery, classifying injuries as: Grade I (confined to the tunica intima), Grade II (intramural hematoma), Grade III (pseudoaneurysm), or Grade IV (complete artery wall rupture). In view of the consolidation of endovascular treatment in surgical practice and the areas of uncertainty that remain with relation to this procedure,^5^ the objective of this study is to describe the experience of a specialist trauma center in Curitiba (Paraná, Brazil) with endovascular treatment of 16 cases of blunt trauma thoracic aortic injury.

## MATERIALS AND METHODS

This is a retrospective, descriptive study, conducted by review of electronic medical records for patients who had suffered blunt thoracic aorta trauma from 2012 to 2019 and were admitted to a hospital specialized in caring for multiple trauma patients, where they were treated using endovascular methods. Qualitative analyses were conducted of epidemiological variables (sex, age, trauma mechanism, and additional injuries), and of variables related to the aortic trauma (injury grade according to the Society for Vascular Surgery classification, material used for endovascular treatment, oversizing, time elapsed between trauma and endovascular treatment, imaging follow-up, and complications associated with the method).

## RESULTS

A total of 16 patients were included in the study, the majority of whom were male (n = 14). Mean age was 37 years and all patients were traffic accident victims, 12 were victims of accidents involving collisions between vehicles (75%) — half of whom were motorcyclists and the other half of whom were automobile drivers. Three patients (18.7%) had been run over and one patient had been in a car that had rolled over (Table 1).

**Table 1:** Epidemiological characteristics of the patients.

**Epidemiological characteristics**	
Gender	
Male	14 (87.5%)
Female	2 (12.5%)
Age (mean)	37 years
Mechanism of trauma	
Collision	12 (75%)
Run over	3 (18.7%)
Vehicle rolled over	1 (6.3%)

**Figure 1:**
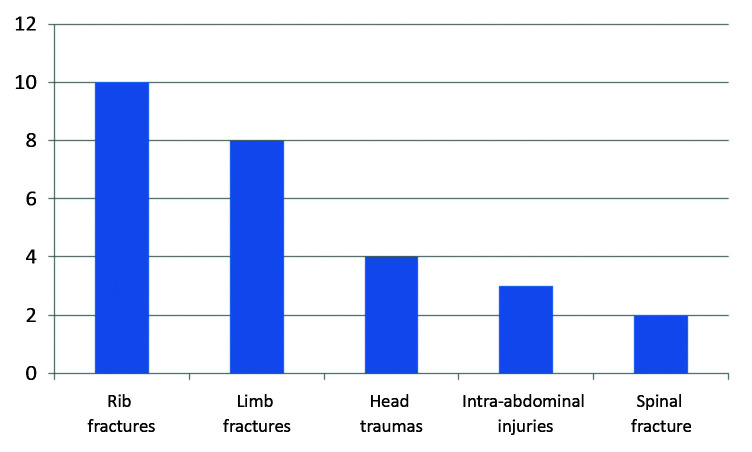
Graph of most common concurrent injuries.

The majority of patients (87.5%) presented with other injuries in addition to the blunt aortic trauma. The principal sites involved were the chest, lower limbs, and head (Figure 1). Ten (62.5%) patients had fractured ribs, associated or not with hemo/pneumothorax. Eight patients (50%) had limb fractures, the majority of which were lower limbs. Four patients (25%) had traumatic brain injuries (TBI). Three patients (18.75%) had intra-abdominal injuries, but only one of these was treated surgically – the other two were prescribed conservative treatment. Two patients (12.5%) had spinal fractures, one of whom had a complete spinal cord injury.

**Figure 2:**
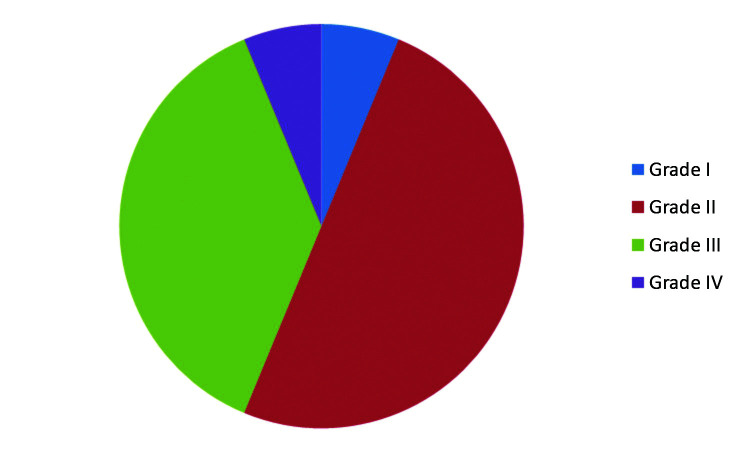
Graph showing distribution of cases by grade of injury.

**Figure 3:**
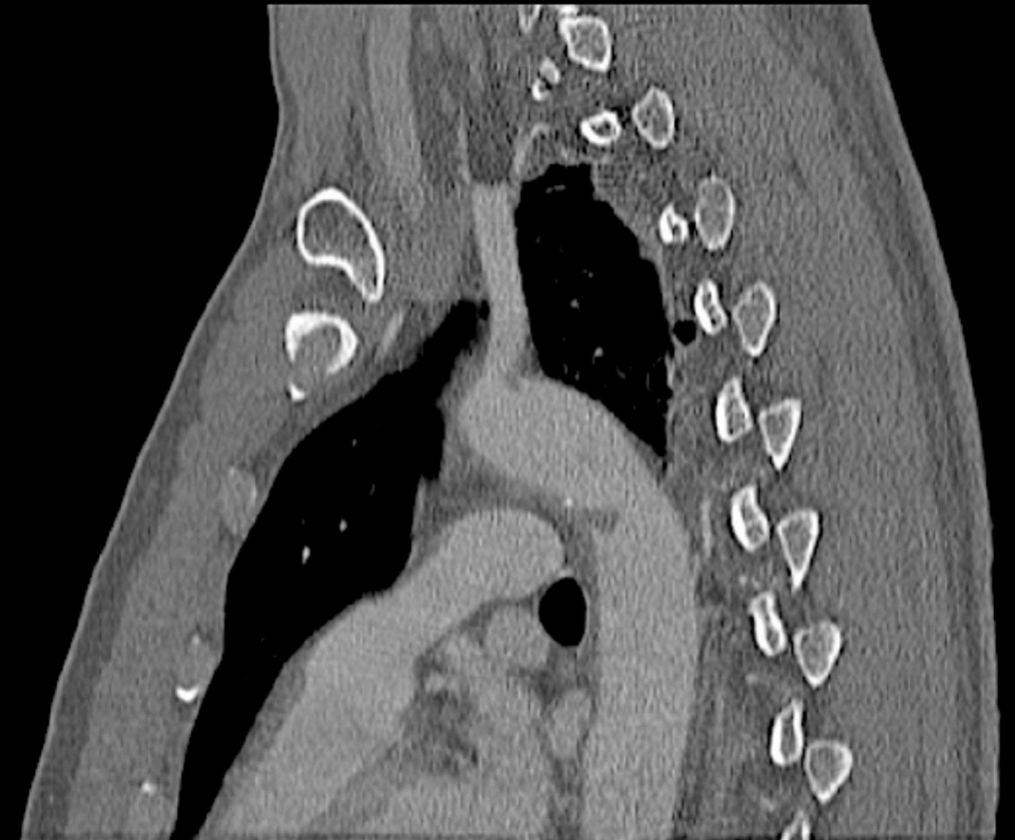
Diagnostic angiotomography of Grade I aortic injury – case 3.

**Figure 4:**
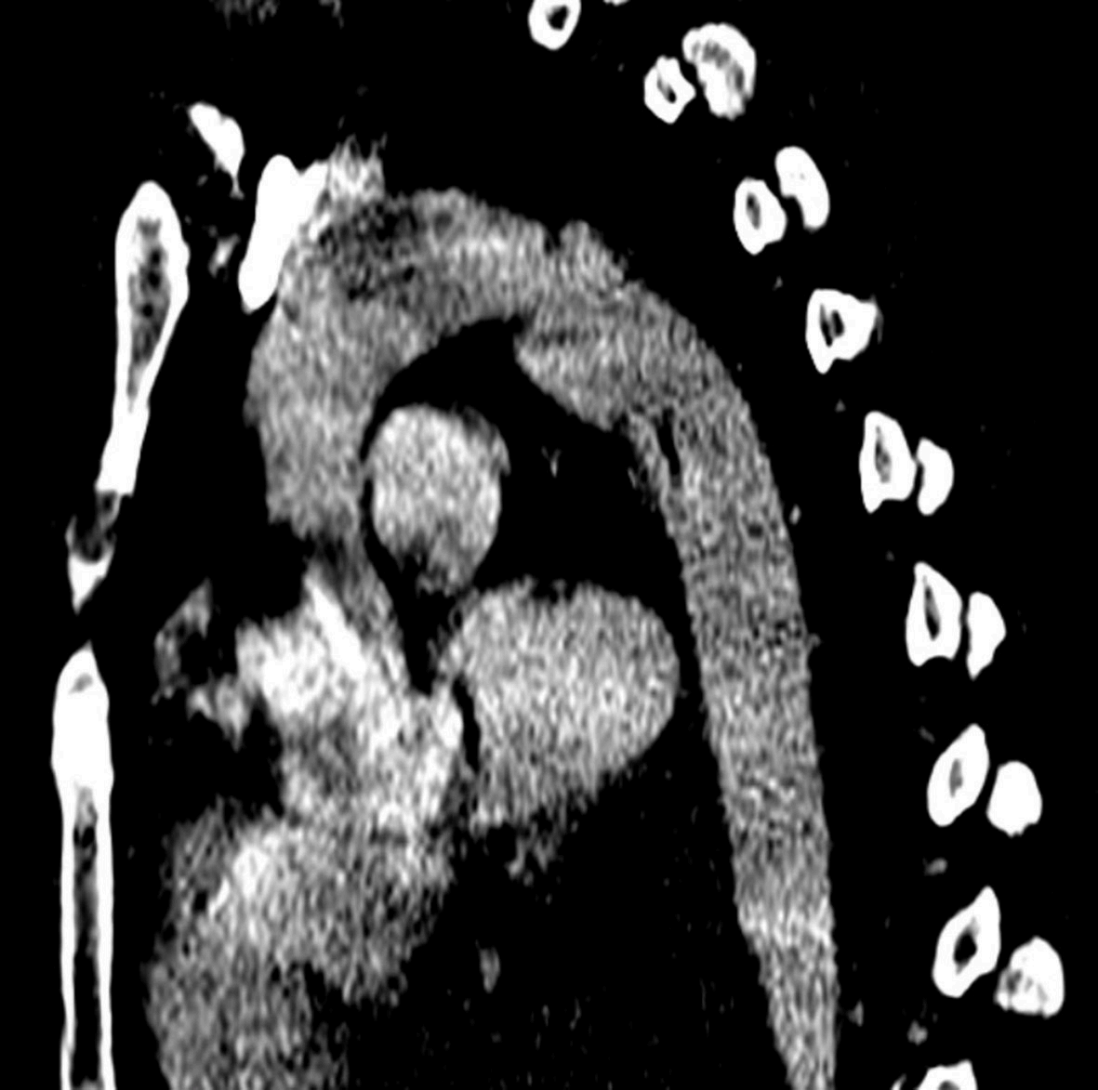
Diagnostic angiotomography of Grade II aortic injury – case 9.

**Figure 5:**
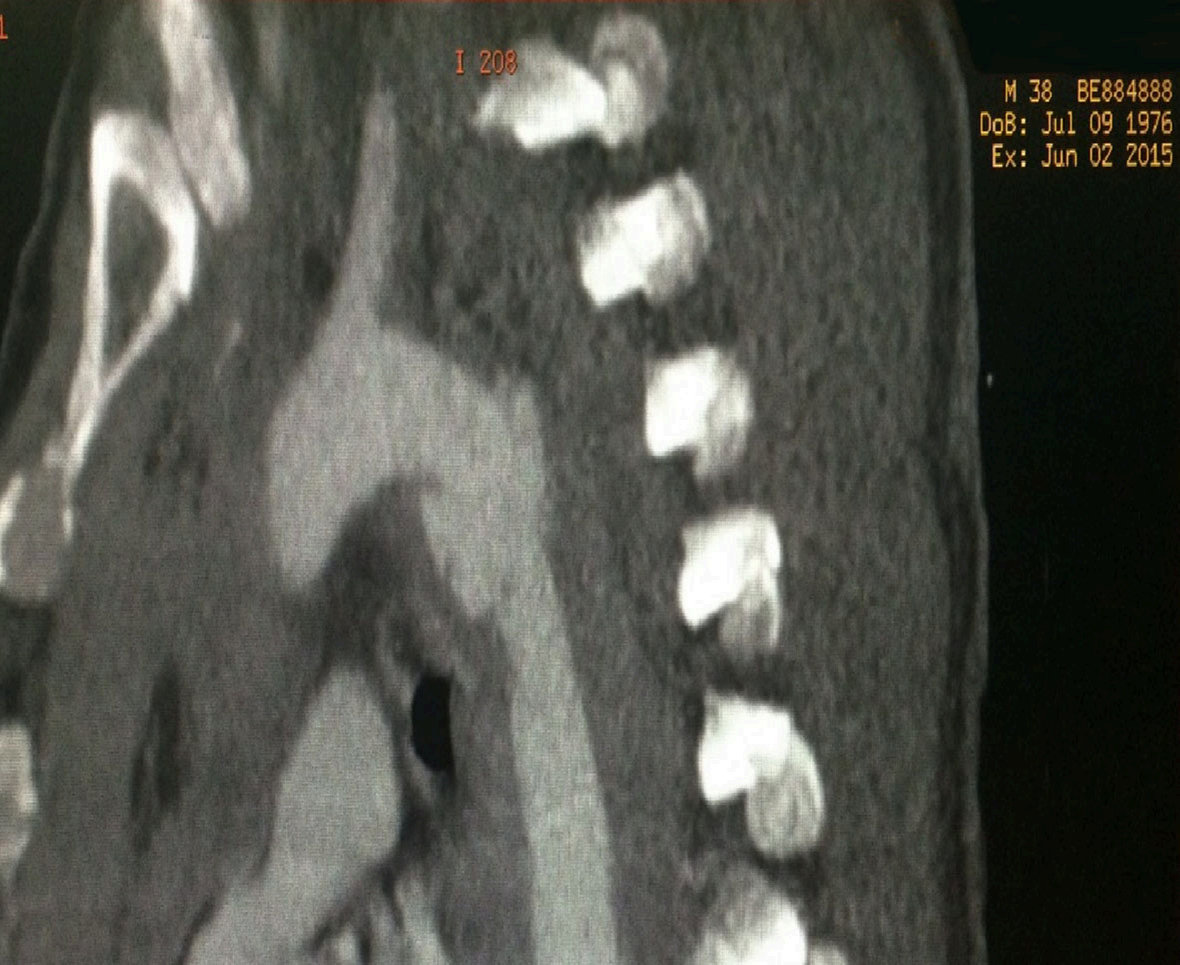
Diagnostic angiotomography of Grade III aortic injury – case 10.

**Figure 6:**
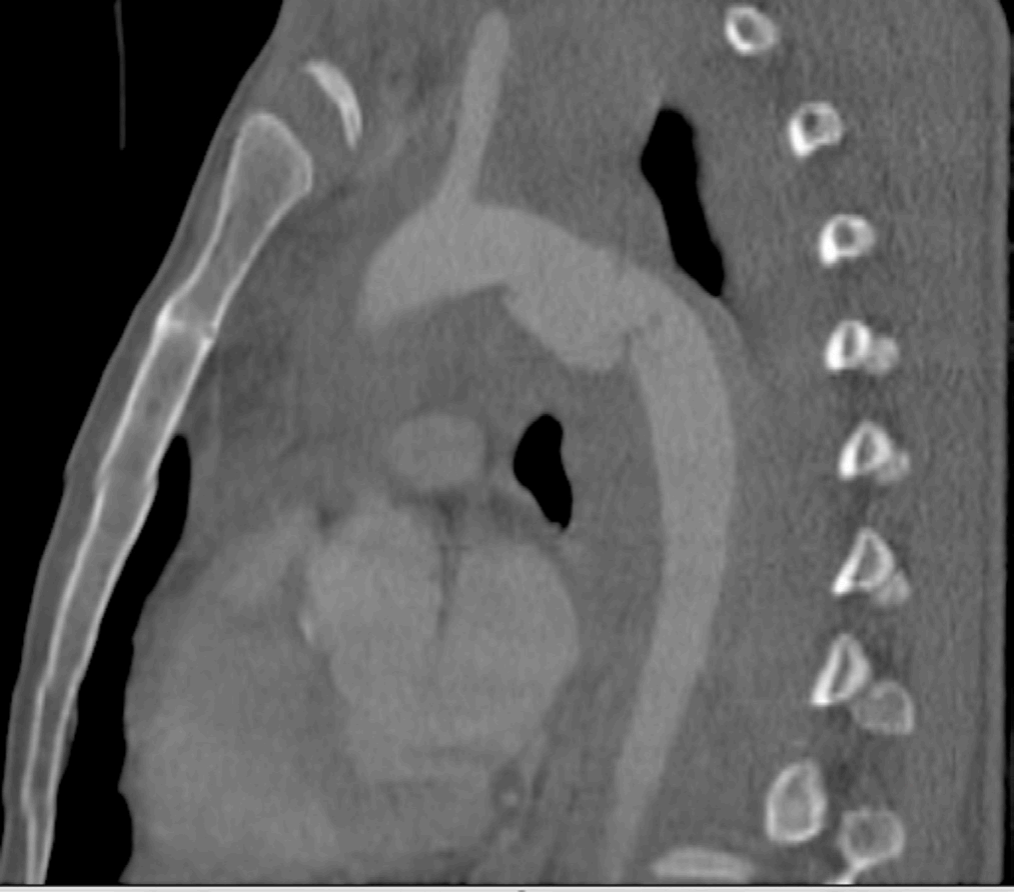
Diagnostic angiotomography of Grade IV aortic injury –case 6.

Aortic injuries ranged from Grades I to IV, as follows: Grade I – 1 case; Grade II – 8 cases; Grade III – 6 cases; and Grade IV – 1 case (Figure 2). All patients underwent endovascular treatment with endograft deployment. Mean time elapsed from trauma to treatment was 71.4 hours (range: 6 hours - 288 hours). Figures 3, 4, 5, and 6 illustrate, respectively, grade I to grade IV injuries seen in patients from this series.

**Figure 7:**
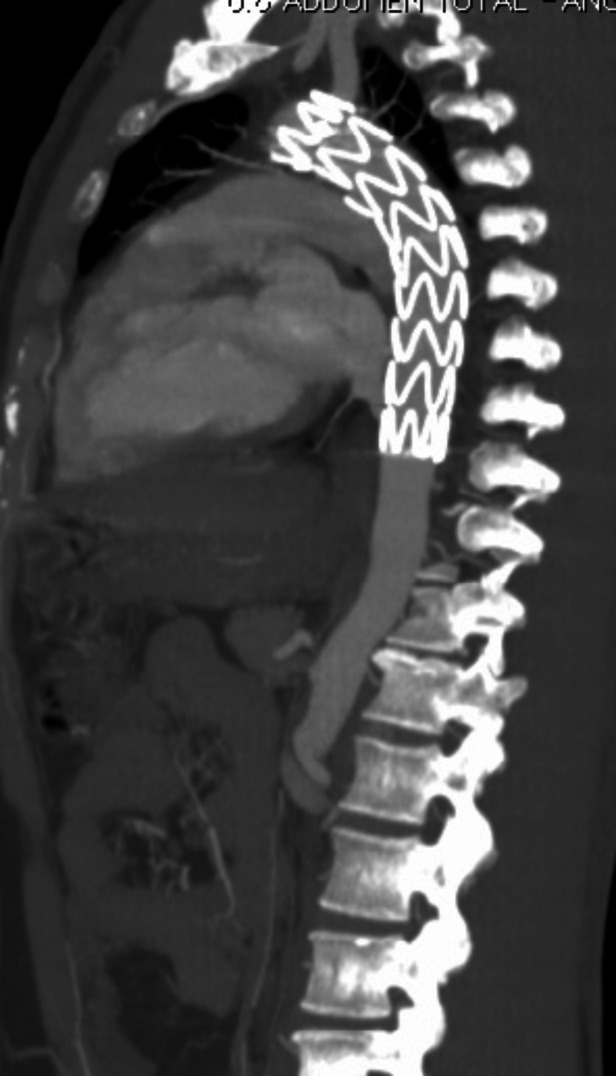
Control angiotomography 30 days after endovascular treatment – case 2.

**Figure 8:**
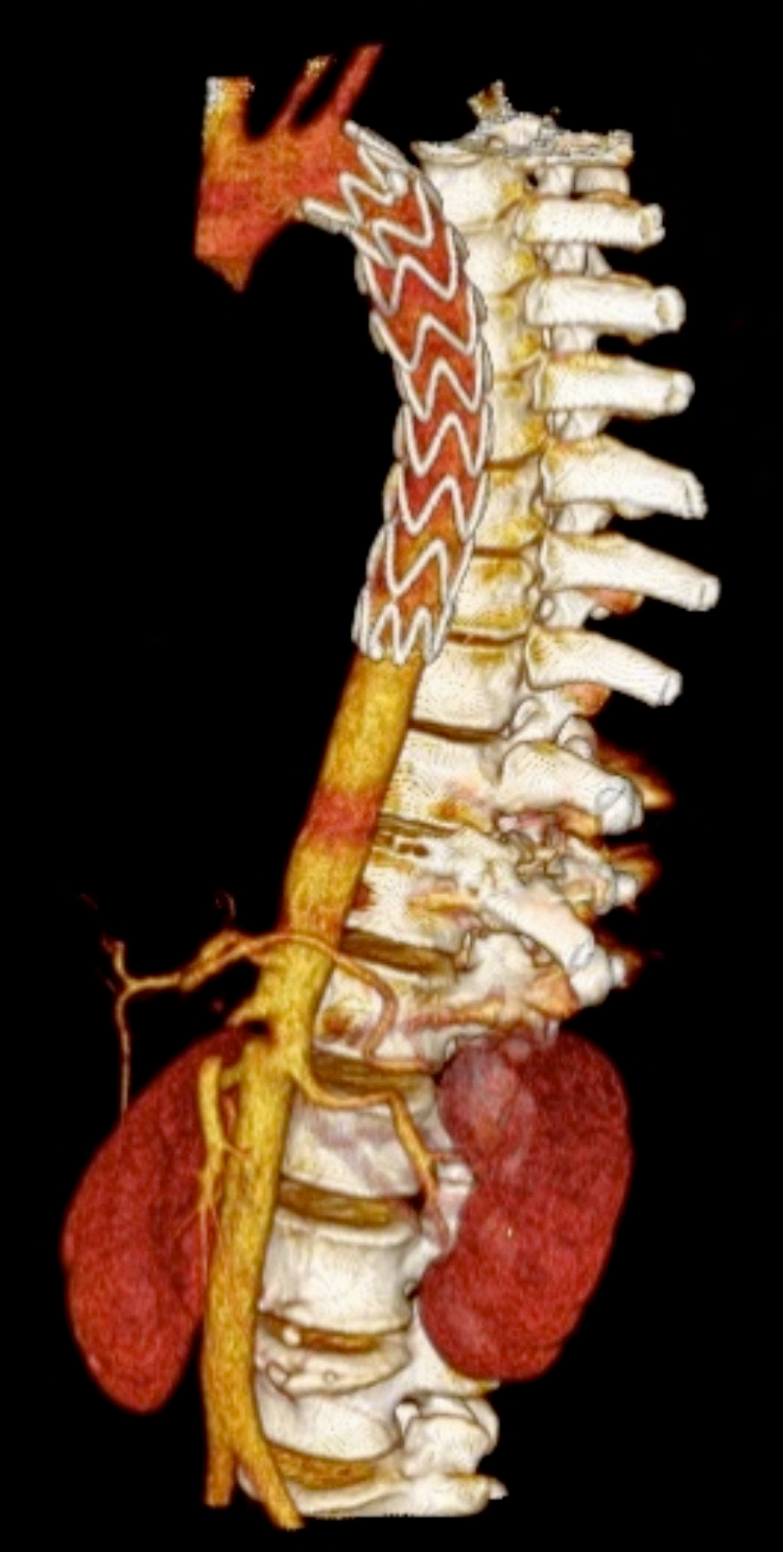
Control angiotomography 6 months after endovascular treatment – case 5.

With relation to the materials chosen for endovascular treatment of aortic injuries, Valiant Captivia® stent grafts (Medtronic, Minneapolis, United States) were used in eight patients; Endurant II® stent grafts (Medtronic) were used in four patients, and a Apolo Thoracic stent graft (NANO®, Santa Catarina, Brazil), Gore Tag® endograft (L. Gore & Associates, Inc., Delaware, United States), a Cook Zenith® endovascular graft (Cook Group Inc., Indiana, United States), and an Endurant® stent graft (Medtronic) were each used in one patient. In two patients, stents were deployed in the left subclavian artery and in both cases the device used was an Advanta V12® (Getinge AB, Getinge, Sweden). Figures 7 and 8 show images from control examinations of two patients after endograft deployment. Data on proximal and distal diameters, oversizing, and the time elapsed between trauma and treatment are listed in Table 2.

**Table 2:** Description of cases by grade of injury, dimensions of the aorta, material utilized, oversizing, interval elapsed between trauma and treatment, and surgical complications.

	**Grade**	**Proximal diameter**	**Distal diameter**	**Material**	**Oversizing**	**Interval in hours - trauma to treatment**	**Surgical complications**
Case 1	III	21	16	Endurant® (Medtronic, Minneapolis, United States), 24 × 82 mm	14%	24	None
Case 2	II	26	23	Valiant Captivia® (Medtronic), 30 × 150 mm	15%	288	None
Case 3	I	23	23	Valiant Captivia® (Medtronic), 26 × 86 mm	13%	24	None
Case 4	II	30	30	Valiant Captivia® (Medtronic), 34 × 12 cm, + fenestration for left subclavian artery: Advanta-V12® stent (Getinge AB, Getinge, Sweden), 10 × 38 cm	13%	60	Dissection progressed
Case 5	III	24	18	Valiant Captivia® (Medtronic), 28 × 157 mm	16%	36	None
Case 6	IV	20	19	Endurant II® (Medtronic), 24 × 82 mm (extension branch)	20%	36	None
Case 7	II	22	20	Apolo Thoracic stent graft (NANO®, Santa Catarina, Brazil), 25 × 120 mm	13%	72	None
Case 8	II	20	18	Valiant Captivia® (Medtronic), 24 × 150 mm	14%	34	Death – unrelated to aortic trauma
Case 9	II	24	21	Endurant II® (Medtronic), 28 × 82 mm	16%	6	None
Case 10	III	23	20	Valiant Captivia® (Medtronic), 28 × 157 mm	21%	144	None
Case 11	II	22	18	Valiant Thoracic® (Medtronic) with manual fenestration, 26 × 164 mm	18%	72	Late type II endoleak
Case 12	II	22	20	Gore Tag® (L. Gore & Associates, Inc., Delaware, United States), 26 × 100 mm	18%	24	None
Case 13	III	19	16	Valiant Captivia® (Medtronic), 22 × 100 mm	15%	12	None
Case 14	II	22	19	Endurant II® (Medtronic), 26 × 130 mm	18%	48	None
Case 15	III	28	24	Cook Zenith® (Cook Group Inc., Indiana, United States), 34 × 34 × 159 mm	21%	72	None
Case 16	III	28	23	Valiant® (Medtronic) 34 × 34 × 150 mm	21%	192	Death - unrelated to aortic trauma

Two of the patients in the study sample died. One died 6 days after the endograft was implanted and the other died 12 days after treatment. Both of these patients died from causes unrelated to the thoracic aorta trauma: both from septic shock, one with an abdominal infection and the other with a pulmonary infection.

During follow-up, just two patients exhibited complications related to endovascular treatment. One patient had a type II endoleak and in another patient the dissection continued to progress. In both cases, conservative treatment was chosen and the patients had no further complications.

## DISCUSSION

The majority of trauma victims are young patients of working age, whether because they are more exposed to risk or because of lifestyle habits involved. The majority of these patients were young and all the cases in the series described here were caused by traffic accidents.^1^^,^^3^ Aortic injuries are usually caused by high-energy trauma mechanisms and are related with simultaneous involvement of other organs. Chest injuries, such as fractured ribs, with or without hemo/pneumothorax, and limb fractures are frequently seen in association with aortic injuries.^5^^,^^9^

Blunt thoracic aorta traumas are fatal for the majority of patients. It is estimated that more than 80% of victims die before they arrive at hospital, while 50% of those patients who arrive alive will die within 24 hours of hospital admission. Early diagnosis is essential for appropriate management of these patients, both to avoid exacerbation of the injury and in order to enable planning of appropriate treatment.^10^^,^^11^ It should be remembered that many injuries are underdiagnosed in emergency, especially in the absence of severe hemodynamic repercussions. Correlations with the mechanism of trauma and the energy level involved are therefore essential to arouse suspicion of injuries.

With relation to surgical repair, endovascular techniques are more effective and practical tools for treatment of blunt trauma aortic injuries in hemodynamically stable patients than conventional surgical approaches.^4^ This tendency is growing because of the lower rates of mortality, morbidity, and postoperative complications linked with endovascular methods when compared with open surgery.^4^^,^^5^ Analysis of a sample of 3,774 patients who had suffered blunt thoracic aortic injury over a 10 year period revealed a significant trend for reduced use of conventional surgery and an increasing tendency to choose endovascular techniques, in addition to a 50% reduction in mortality among patients treated with these techniques.^5^

DuBose et al.^4^ conducted a multicenter study with 382 patients with aortic injuries caused by thoracic contusion managed with one of three approaches: conservative, open surgery, or endovascular treatment with aortic endografts. When compared, the authors demonstrated that mortality was lowest in the group treated with endovascular methods, with rates of 34.4, 19.7, and 8.6% respectively.^4^ Recently, a study of 3,628 patients who underwent endovascular treatment or open surgery demonstrated with multivariate logistic regression analysis that open surgery was an independent risk factor for mortality in blunt aortic trauma patients (odds ratio [OR] 1.63, confidence interval [CI] 95% 1.19-2.23, p < 0.05). Furthermore, endovascular treatments was associated with shorter length of both entire hospital stay and stay in the intensive care unit (ICU) and also a lower rate of operative complications.^6^ It is because of this consolidation of endovascular methods for treatment of blunt trauma thoracic aorta injuries that this was the method of choice for treatment of all of the patients in the present case series.

The decision on the most appropriate treatment depends on the extent of the aortic injury caused by the blunt trauma and also on the patient’s hemodynamic stability. According to the Azizzadeh classification used by the Society for Vascular Surgery,^7^^,^^8^ the patients in the present study had injury grades varying from I to IV. Surgical treatment is proposed for cases with grade II, III, or IV injuries, although there are reports in the literature of nonoperative management in some cases of traumatic pseudoaneurysm.^8^^,^^9^ However, there is no consensus on which injuries are indicated for surgical treatment, although there are reports of good results with conservative treatment for those whose aortic injuries are grade I or II.^4^^,^^10^

In the multicenter RESCUE trial (thoracic endovascular aortic repair for blunt thoracic aortic injury),^7^ approximately 18% of the patients treated had grade I aortic injuries. Among these cases, the criteria for indication of treatment were primarily concomitant intracranial injuries, hypotension requiring vasoactive drugs, extensive intimal injuries, or concomitant injuries. Nevertheless, indications for surgical treatment in these cases remain controversial and there is also a possibility of conservative management with frequent control imaging exams. In the present study, just one patient was classified as grade I (case 3), in whom endovascular treatment was chosen regardless. Experience with endovascular treatment of these injuries, the presence of concomitant injuries (fractured ribs and pneumothorax), and uncertainty with relation to the possibility of adequate follow-up with control examinations were the determinant factors in deciding to treat this patient.

Although endovascular treatment is a new alternative to open surgery and offers a less invasive approach for patients in a critical state, the technique can result in significant complications related to endografts (endoleaks and migration or rupture of devices) or in situations of ischemia caused by embolic events (stroke, paraplegia, and ischemic spinal cord injury).^12^ However, over recent years, it has been observed that improvements in surgical techniques and endovascular devices have led to significant reductions in the incidence of complications related to endovascular treatment.^3^

Studies show reductions in paraplegia rates^3^^,^^4^^,^^13^ and lower risk of postoperative complications (such as acute renal failure^14^ and acute respiratory distress syndrome^6^) in patients with traumatic aortic injuries treated with endovascular methods when compared with patients treated with open surgery. The risk of stroke in patients treated with endovascular methods was comparable to the risk linked to open surgery or there was no statistically significant difference between the methods.^3^^,^^6^

Although the majority of studies indicate the endovascular method’s superiority over open surgery in terms of rates of mortality and complications for patients with blunt aortic traumas, the medium and long term results and the durability of devices are not yet known.^13^^,^^15^

The prospective RESCUE^13^ study is currently evaluating the results of endovascular treatment of these patients over a 5-year follow-up period. At each visit, patients undergo clinical examination and an imaging exam (angiotomography or magnetic resonance). After 1 year of follow-up, no cases were observed of paraplegia, paraparesis, or stroke. No adverse events related to the device were detected. With regard to adverse events related to the procedure, 16% of the patients exhibited ischemic events in the left upper limb caused by intentional obstruction of the subclavian artery (8%) or injuries related to the puncture site (8%). Revascularization of the left subclavian artery was needed in 8% of the patients. Mortality during the study period was 12% and 8% of the patients died within the first 30 days of follow-up.

García Reyes et al.^15^ conducted a study to investigate the long-term results of blunt thoracic aorta trauma patients treated using endovascular techniques. Mean follow-up of the participants was 98 months and the most common complication observed was intragraft thrombus (20%). Just 9% required reintervention. In half of these, revascularization of the left subclavian artery was performed and in the other half aortic reinterventions were conducted because of endoleak or occlusive thrombus in the endografts. All reinterventions were successful and no additional complications were reported. None of the patients suffered paraplegia or neurological damage and there were no deaths during the perioperative period or over the course of follow-up.

In the present study, just two patients exhibited complications related to the procedure. In these cases, expectant management was adopted and both cases proceeded with no additional intercurrent conditions. Control angiotomography is conducted in outpatients follow-up at 30 to 90 days after the procedure and at 6 months. Angiotomography is then performed once more at 36 months (3 years) and 60 months (5 years) after the procedure.

Another important detail related to this treatment is the degree of graft oversizing used in these patients, since the majority of cases were young people with a previously healthy thoracic aorta. Since the endografts were not originally manufactured for treatment of traumatic aortic injuries, these devices have structures compatible with larger diameter aortas, as seen in aneurysmal disease. As a result, excessive oversizing is very often inevitable in patients with blunt trauma aortic injuries, because of a lack of endografts of the correct size.^15^ Current recommendations in the literature limit maximum oversizing to 20%.^16^ The size of the endografts used and their positioning are determinant factors in the post-treatment results. García Reyes et al.^15^ observed that patients who exhibited complications related to endografts had greater oversizing than a subset free from complications (p = 0.0007).^15^

The majority of devices currently available are not specifically designed for traumatic injuries to the aorta, and it is difficult to find the smaller sizes that would be ideal for younger patients.^16^ This obliges many surgeons to implant endografts with greater oversizing, as the only viable option for endovascular treatment. However, excessive oversizing can be related to type I endoleak and kinking and collapse of endografts.^17^

Some of the most important endoprostheses approved for use are the Valiant® (Medtronic), the C-TAG® (L. Gore & Associates, Inc.), and the TX2® (Cook Group Inc.). However, consideration should be given to the availability of these materials at services and also to the anatomy of the patient, in order to choose the most appropriate device. An endoprosthesis with greater conformability reduces the spring-back force exerted and also reduces the likelihood of future endoleaks or endograft collapse, which can make them more suited to these cases.^17^ The profiles of thoracic stent grafts vary, on average, from 18-24Fr, which can very often be incompatible with the diameter of the femoral arteries for delivery of thoracic endoprostheses, so low profile endoprostheses with smaller calibers are therefore preferable.

The diameter of the aorta proximal of the injury varied from 19 mm to 30 mm, where the largest diameter was seen in a 67-year-old patient who possibly had prior chronic dissection (case 4 – Table 2). In turn, oversizing varied from 11 to 21%. The initial objective was to limit oversizing to the region of 10-15%, but, as explained earlier, there were variations related to availability of materials, so that the smallest diameter available at the time was often used.

New studies will tend to establish the best time for endovascular treatment of patients with blunt trauma aortic injuries. Some studies have already shown that there is an advantage from late intervention (after 24 hours) in relation to early intervention (in the first 24 hours), even in patients with very severe additional injuries.^18^ Marcaccio et al.^18^ demonstrated in a sample of 507 patients that later intervention was associated with lower mortality rates (5.4%) than early treatment (11.9%). Early intervention was also associated with a higher risk of mortality in a multivariate logistic regression analysis (OR 2.39; 95%CI 1.01-5.67; p = 0.047).

Although to date there are no randomized and controlled studies comparing endovascular treatment with open surgery for patients with aortic injuries caused by blunt trauma, there has been significant improvement in mortality and morbidity rates as endovascular approaches have replaced conventional open surgery at the majority of trauma centers.^12^^,^^18^

## CONCLUSIONS

The endovascular method is a feasible alternative for treatment of thoracic aorta injuries caused by blunt trauma, as supported both by the literature and by the service’s experience. Applicability and lower morbidity and mortality are factors to be taken into account when choosing the endovascular technique. Randomized, controlled studies, or at least long-term follow-up of patients, are needed to provide additional evidence in support of this method as a treatment for this type of injury.
