# Supplementary material for: Endovascular treatment of traumatic dissection of the thoracic aorta – series of 16 cases
Source: J Vasc Bras. 2020 Nov 16;19:e20200074. doi: 10.1590/1677-5449.200074 (PMC8218017; doi:10.1590/1677-5449.200074)
Supplement: Translation

# Translation: Tratamento endovascular de dissecção traumática de aorta torácica – série de 16 casos

**Authors:** Lucas Mansano Sarquis, Wilson Michaelis, Antonio Lacerda Santos, Cristiano Silva Pinto, Rogerio Akira Yokoyama, Erick Fernando Seguro, Antonio Luiz da Costa Martins, Vinicius Belas do Vale

**Affiliations:** 1 Hospital do Trabalhador – HT, Serviço de Cirurgia Vascular, Curitiba, PR, Brasil.; 2 Hospital Universitário Evangélico Mackenzie – HUEM, Serviço de Cirurgia Vascular, Curitiba, PR, Brasil.; 3 Universidade Federal do Paraná – UFPR, Faculdade de Medicina, Curitiba, PR, Brasil.

**Keywords:** aorta torácica, lesões, doenças da aorta, procedimentos endovasculares

## Abstract

**Contexto:**

As lesões de aorta nos traumatismos torácicos fechados possuem alta mortalidade pré-hospitalar e no serviço de emergência. O tratamento endovascular é um método de escolha para o tratamento dessas lesões; entretanto, muitos resultados em relação a essa abordagem permanecem desconhecidos.

**Objetivos:**

O objetivo deste trabalho é descrever a experiência no tratamento endovascular de lesões traumáticas de aorta torácica em um centro de referência em trauma.

**Métodos:**

Este trabalho trata-se de estudo descritivo realizado através da revisão de prontuários eletrônicos de pacientes vítimas de trauma contuso de aorta torácica, atendidos em um hospital de referência em trauma na cidade de Curitiba (Paraná, Brasil).

**Resultados:**

Dezesseis pacientes foram incluídos no estudo. Todos os pacientes foram vítimas de acidente de trânsito, sendo que 75% dos acidentes ocorreram por colisão entre veículos. As lesões de aorta variaram de Grau I a IV, e a maioria dos pacientes apresentou lesão de grau II (50%). Todos os pacientes foram submetidos a terapia endovascular com implante de endoprótese sendo realizado em média 71 horas após o trauma. Dois pacientes evoluíram a óbito, porém de causas não relacionadas à lesão de aorta. Durante o seguimento, apenas dois pacientes apresentaram complicações (*endoleak* e progressão da dissecção).

**Conclusões:**

O método endovascular é uma alternativa viável no tratamento de lesões de aorta torácica por trauma contuso. São necessários estudos randomizados e controlados a fim de reforçar a indicação desse método como terapia para esse tipo de lesão.

## INTRODUÇÃO

O trauma fechado de aorta torácica é a segunda causa de óbito em pacientes vítimas de trauma e apresenta taxa de mortalidade pré-hospitalar de 80%, ficando atrás apenas do traumatismo craniano^1^^,^^2^. O principal fator de risco para lesão de aorta torácica em trauma contuso é a desaceleração brusca, sendo os mecanismos de trauma mais prevalentes as colisões por automóveis (70%), acidentes com motocicletas, quedas de outro nível e atropelamentos^3^. Esse tipo de lesão está envolvido em um terço dos casos de morte provocada por colisões entre automóveis^4^.

A maioria dos pacientes vítimas de trauma contuso de aorta torácica são adultos jovens em idade economicamente ativa e apresentam acometimento multissistêmico^1^, com lesões associadas principalmente em crânio, abdome e/ou membros inferiores^5^. O tratamento cirúrgico convencional pode agregar maior risco para esses pacientes politraumatizados e, diante disso, o tratamento endovascular surge como uma alternativa promissora para a abordagem de lesões que acometem a aorta torácica em trauma fechado^1^.

Nas últimas duas décadas, houve uma importante evolução no tratamento dos pacientes que permanecem vivos até receberem atendimento hospitalar. Isso ocorreu graças ao desenvolvimento dos sistemas pré-hospitalares, à capacitação das equipes de emergência e ao aumento dos recursos diagnósticos, como tomografia computadorizada (TC) e métodos endovasculares^4^^,^^5^. Atualmente, o tratamento endovascular se tornou o método de eleição para doentes estáveis hemodinamicamente com lesão da aorta torácica por trauma fechado, além de se associar a menores taxas de mortalidade quando comparado ao tratamento cirúrgico convencional^6^.

As injúrias provocadas à aorta pelo trauma contuso de tórax vão desde lacerações na camada íntima do vaso até a completa ruptura de sua parede^7^^,^^8^, sendo a principal porção acometida o ligamento arterioso, uma região imediatamente distal à artéria subclávia esquerda por ser uma região de transição entre o arco aórtico (relativamente móvel) e a aorta descendente (mais fixa)^2^.

A classificação mais utilizada para lesões traumáticas de aorta torácica é a proposta por Khoynezhad et al.^7^ e adotada pela *Society for Vascular Surgery*, que classifica as lesões em: Grau I (confinada à camada íntima), Grau II (hematoma intramural), Grau III (pseudoaneurisma) e Grau IV (rotura completa da parede arterial). Diante da consolidação do tratamento endovascular na prática cirúrgica e das incertezas que ainda existem acerca desse procedimento^5^, o objetivo deste trabalho é descrever a experiência de um centro de trauma referência em Curitiba (Paraná, Brasil) no tratamento endovascular de 16 casos de lesão em aorta torácica por trauma contuso.

## MATERIAIS E MÉTODOS

Trata-se de estudo descritivo, retrospectivo, realizado através da revisão de prontuários eletrônicos de pacientes vítimas de trauma contuso de aorta torácica, atendidos num hospital referência em atendimento ao paciente politraumatizado, admitidos entre 2012 e 2019 que foram submetidos ao tratamento endovascular. Foi realizada avaliação qualitativa de variáveis epidemiológicas (sexo, idade, mecanismo do trauma, lesões associadas), bem como das variáveis relacionadas ao trauma de aorta (grau da lesão de acordo com a classificação da *Society for Vascular Surgery*, material utilizado no tratamento endovascular, *oversizing*, tempo decorrido entre o trauma e o tratamento endovascular, seguimento com imagem e complicações associadas ao método).

## RESULTADOS

Foram incluídos 16 pacientes no estudo, sendo a maioria do sexo masculino (n = 14). A média de idade foi de 37 anos e todos os pacientes foram vítimas de acidente de trânsito, sendo que 12 acidentes ocorreram por colisão entre veículos (75%) — sendo metade desses pacientes condutores de motocicletas e a outra metade, condutores de automóveis —, três pacientes (18,7%) foram vítimas de atropelamento e um paciente foi vítima de capotamento de automóvel (Tabela 1).

**Tabela 1:** Características epidemiológicas dos pacientes.

**Características epidemiológicas**	
Gênero	
Masculino	14 (87,5%)
Feminino	2 (12,5%)
Idade (média)	37 anos
Mecanismo de trauma	
Colisão	12 (75%)
Atropelamento	3 (18,7%)
Capotamento	1 (6,3%)

A maior parte dos pacientes (87,5%) apresentou lesões associadas ao trauma contuso de aorta. Os principais sítios foram tórax, membros inferiores e crânio (Figura 1). Dez (62,5%) pacientes apresentaram fraturas de arcos costais, associadas ou não com hemo/pneumotórax. Oito pacientes (50%) apresentaram fraturas de membros, sendo a maior parte deles de membros inferiores. Quatro pacientes (25%) apresentaram traumatismo cranioencefálico (TCE). Três pacientes (18,75%) apresentaram lesão intra-abdominal, porém, apenas um foi submetido a tratamento cirúrgico, sendo os demais submetidos a tratamento conservador. Dois pacientes (12,5%) apresentaram fratura de coluna, sendo um deles com lesão raquimedular completa.

**Figura 1:** Gráfico com as principais lesões associadas.

As lesões de aorta variaram de Grau I a IV: Grau I – 1 caso; Grau II – 8 casos; Grau III – 6 casos; Grau IV – 1 caso (Figura 2). Todos os pacientes foram submetidos à terapia endovascular com implante de endoprótese. O tratamento ocorreu após um tempo médio 71,4 horas do episódio de trauma (o tempo mínimo foi 6 horas e o tempo máximo foi 288 horas). As Figuras 3, 4, 5 e 6 representam os graus de lesão, de grau I a grau IV, respectivamente, identificados na presente casuística.

**Figura 2:** Gráfico com a divisão dos casos de acordo com o grau da lesão.

**Figura 3:** Angiotomografia diagnóstica com lesão de aorta Grau 1 – caso 3.

**Figura 4:** Angiotomografia diagnóstica com lesão de aorta Grau 2 – caso 9.

**Figura 5:** Angiotomografia diagnóstica com lesão de aorta Grau 3 – caso 10.

**Figura 6:** Angiotomografia diagnóstica com lesão de aorta Grau 4 – caso 6.

Em relação ao material escolhido no tratamento endovascular da lesão de aorta, a endoprótese Valiant Captivia® (Medtronic, Minneapolis, EUA) foi utilizada em oito pacientes; a prótese Endurant II® (Medtronic) foi utilizada em quatro pacientes e as próteses Apolo Reta Torácica (NANO Endoluminal®, Santa Catarina, Brasil), Gore Tag® (L. Gore & Associates, Inc., Delaware, EUA), Cook Zenith® (Cook Group Inc., Indiana, EUA) e Endurant® (Medtronic) foram utilizadas uma vez cada uma. Em dois pacientes, foram colocadas endopróteses na artéria subclávia esquerda, sendo que em ambos os casos o material utilizado foi Advanta V12® (Getinge AB, Getinge, Suécia). As Figuras 7 e 8 representam os exames de controle de dois pacientes após o implante da endoprótese. Os dados relacionados a diâmetro proximal, distal, *oversizing* e intervalo entre trauma e tratamento estão descritos na Tabela 2.

**Figura 7:** Angiotomografia de controle em 30 dias após o tratamento endovascular – caso 2.

**Figura 8:** Angiotomografia de controle em 6 meses após o tratamento endovascular – caso 5.

**Tabela 2:** Descrição dos casos de acordo com o grau, dimensões da aorta, material utilizado, *oversizing*, intervalo entre trauma e tratamento e as complicações cirúrgicas.

	**Grau**	**Diâmetro proximal**	**Diâmetro distal**	**Material**	***Oversizing***	**Intervalo em horas - trauma e tratamento**	**Complicações cirúrgicas**
Caso 1	III	21	16	Endurant® (Medtronic, Minneapolis, EUA) 24 × 82 mm	14%	24	Ausentes
Caso 2	II	26	23	Valiant Captivia® (Medtronic) 30 × 150 mm	15%	288	Ausentes
Caso 3	I	23	23	Valiant Captivia® (Medtronic) 26 × 86 mm	13%	24	Ausentes
Caso 4	II	30	30	Valiant Captivia® (Medtronic) 34 × 12 cm + fenestração da artéria subclávia esquerda: stent Advanta-V12® (Getinge AB, Getinge, Suécia) 10 × 38 cm	13%	60	Progressão da dissecção
Caso 5	III	24	18	Valiant Captivia® (Medtronic) 28 × 157 mm	16%	36	Ausentes
Caso 6	IV	20	19	Endurant II® (Medtronic) 24 × 82 mm (ramo extensor)	20%	36	Ausentes
Caso 7	II	22	20	Apolo Reta Torácica (NANO®, Santa Catarina, Brasil) 25 × 120 mm	13%	72	Ausentes
Caso 8	II	20	18	Valiant Captivia® (Medtronic) 24 × 150 mm	14%	34	Óbito - não relacionado ao trauma de aorta
Caso 9	II	24	21	Endurant II® (Medtronic) 28 × 82 mm	16%	6	Ausentes
Caso 10	III	23	20	Valiant Captivia® (Medtronic) 28 × 157 mm	21%	144	Ausentes
Caso 11	II	22	18	Valiant Torácica® (Medtronic) com fenestração manual 26 × 164 mm	18%	72	*Endoleak* tipo 2 tardio
Caso 12	II	22	20	Gore Tag® (L. Gore & Associates, Inc., Delaware, EUA) 26 × 100 mm	18%	24	Ausentes
Caso 13	III	19	16	Valiant Captivia® (Medtronic) 22 × 100 mm	15%	12	Ausentes
Caso 14	II	22	19	Endurant II® (Medtronic) 26 × 130 mm	18%	48	Ausentes
Caso 15	III	28	24	Cook Zenith® (Cook Group Inc., Indiana, EUA) 34 × 34 × 159 mm	21%	72	Ausentes
Caso 16	III	28	23	Valiant® (Medtronic) 34 × 34 × 150 mm	21%	192	Óbito - não relacionado ao trauma de aorta

Dois pacientes do estudo evoluíram para óbito, sendo que um deles faleceu 6 dias após a colocação da endoprótese e o outro, 12 dias após. Os dois os pacientes faleceram por causas não relacionadas ao trauma de aorta torácica: ambos por choque séptico, um de foco abdominal e outro de foco pulmonar.

Durante o seguimento, apenas dois pacientes apresentaram complicações relacionadas ao tratamento endovascular. Um paciente apresentou *endoleak* tipo II, enquanto, em outro paciente, ocorreu progressão da dissecção, sendo optado pelo tratamento conservador em ambos os casos, os quais evoluíram sem novas intercorrências.

## DISCUSSÃO

Os pacientes jovens em idade economicamente ativa são os principais envolvidos nos traumas, seja pela maior exposição a riscos ou pelos hábitos de vida envolvidos. A maioria dos pacientes eram jovens, sendo acidentes de trânsito responsáveis por todos os casos do presente estudo^1^^,^^3^. As lesões de aorta geralmente estão envolvidas com mecanismos de trauma de alta energia, o que se relaciona com acometimento simultâneo de outros órgãos. As lesões torácicas, como fratura de arcos costais e/ou hemo/pneumotórax, e as fraturas de membros são frequentemente associadas às lesões aórticas^5^^,^^9^.

O trauma contuso de aorta torácica é fatal para a maioria dos pacientes. Estima-se que mais de 80% das vítimas morrem antes de chegar ao hospital, enquanto 50% dos pacientes que sobrevivem morrem nas primeiras 24 horas após a admissão hospitalar. O diagnóstico precoce é essencial para o manejo adequado desses pacientes, tanto para evitar a progressão da lesão como para poder planejar o tratamento adequado^10^^,^^11^. Deve-se atentar para o fato de muitas lesões serem subdiagnosticadas na emergência, especialmente se não há repercussão hemodinâmica grave. Sendo assim, a correlação do mecanismo de trauma e o grau de energia envolvido é essencial para suspeição da lesão.

Em relação à correção cirúrgica, a técnica endovascular para o tratamento das lesões de aorta por trauma contuso é uma ferramenta eficaz e factível para os pacientes estáveis hemodinamicamente em detrimento da abordagem cirúrgica convencional^4^. Essa tendência ocorre devido a menores taxas de mortalidade, morbidade e complicações pós-operatórias que a abordagem endovascular possui quando comparada à cirurgia aberta^4^^,^^5^. Uma amostra de 3.774 pacientes reuniu vítimas de lesão de aorta torácica por trauma contuso ao longo de dez anos e observou uma tendência significante no declínio do uso da cirurgia convencional e um aumento na escolha da técnica endovascular, bem como redução de 50% da mortalidade dos pacientes submetidos a essa técnica^5^.

Em um estudo multicêntrico realizado por DuBose et al.^4^, 382 pacientes vítimas de lesão de aorta por contusão torácica foram manejados em três modalidades terapêuticas: conservadora, cirurgia aberta e tratamento endovascular com endoprótese aórtica. Quando comparadas, os autores demonstraram uma menor mortalidade no grupo tratado de forma endovascular, com taxas de 34,4, 19,7 e 8,6% respectivamente^4^. Recentemente, um estudo envolvendo 3.628 pacientes submetidos a tratamento endovascular e cirurgia aberta demostrou, em uma análise de regressão logística multivariada, que a cirurgia aberta representa um fator de risco independente para mortalidade em pacientes vítimas de trauma contuso de aorta [*odds ratio* (OR) 1.63, intervalo de confiança (IC) 95% 1,19-2,23, p < 0,05]. Ademais, o manejo endovascular se associou a menor tempo de internamento hospitalar total e em unidade de terapia intensiva (UTI), bem como a menor taxa de complicações operatórias^6^. Diante da consolidação do método endovascular para o tratamento de lesões de aorta torácica por trauma contuso, essa abordagem foi o método de escolha para o tratamento de todos os pacientes do estudo.

A escolha do tratamento mais adequado depende da extensão da injúria aórtica causada pelo trauma contuso, bem da estabilidade hemodinâmica do paciente. Utilizando a classificação proposta por Azizzadeh e utilizada pela *Society for Vascular Surgery* 7^,^^8^, os graus de lesão dos pacientes incluídos no presente estudo variaram de I a IV. O tratamento cirúrgico é proposto para os casos com lesões de Grau II, III e IV, embora existam relatos na literatura de tratamento não operatório em alguns casos de pseudoaneurisma traumático^8^^,^^9^. Entretanto, não há consenso sobre quais lesões são indicativas de tratamento cirúrgico, existindo relatos de bons resultados com o tratamento conservador para aqueles que apresentam lesão aórtica Grau I ou II^4^^,^^10^.

No estudo multicêntrico RESCUE (*endovascular treatment of blunt thoracic aortic injuries*)^7^, aproximadamente 18% dos pacientes tratados apresentavam lesões de aorta Grau I. Nesses casos, os critérios para indicação do tratamento foram principalmente lesões intracranianas concomitantes, hipotensão com necessidade de droga vasoativa, lesões extensas de íntima ou lesões concomitantes. Apesar disso, ainda existem controvérsias quanto à indicação de tratamento cirúrgico nesses casos, havendo a possibilidade de tratamento conservador com exames de imagem de controle frequentes. No presente estudo, apenas um paciente foi classificado como Grau I (caso 3), entretanto, foi proposto o tratamento endovascular. A experiência com o tratamento endovascular dessas lesões, assim como a presença de lesões concomitantes (fraturas de arcos costais e pneumotórax) e a incerteza da possibilidade de seguimento com exames de controle de forma adequada foram fatores determinantes para o tratamento desse paciente.

Embora o tratamento endovascular seja uma nova alternativa à cirurgia aberta e ofereça um meio de abordagem menos invasivo aos pacientes que se encontram em um estado crítico, essa técnica pode resultar em complicações importantes relacionadas à endoprótese (*endoleak*, migração ou ruptura do dispositivo) ou em situações de isquemia devido a eventos embólicos [acidente vascular cerebral (AVC), paraplegia, lesão isquêmica da medula]^12^. Entretanto, observou-se que, nos últimos anos, com o aprimoramento da técnica cirúrgica e das endopróteses, houve uma redução significativa na incidência das complicações relacionadas ao tratamento endovascular^3^.

Estudos mostraram redução nas taxas de paraplegia^3^^,^^4^^,^^13^ e menor risco de complicações pós-operatórias, como insuficiência renal aguda^14^ e síndrome de desconforto respiratório agudo^6^, em pacientes com lesões traumáticas de aorta tratados pelo método endovascular quando comparados a pacientes tratados com cirurgia aberta. O risco de AVC em pacientes tratados pelo método endovascular foi comparável ao risco da cirurgia aberta ou não houve diferença estatisticamente significativa entre os métodos^3^^,^^6^.

Embora a maioria dos estudos apontem para uma superioridade do método endovascular sobre a cirurgia aberta, em relação às taxas de mortalidade e de complicações em pacientes vítimas de trauma contuso de aorta, os resultados a médio e longo prazo e a durabilidade do dispositivo ainda são desconhecidos^13^^,^^15^.

O estudo prospectivo RESCUE^13^ avalia atualmente os resultados do tratamento endovascular nesses pacientes através de um período de 5 anos de seguimento. Em cada visita, o paciente é submetido a um exame clínico e um exame de imagem (angiotomografia ou ressonância magnética). Após um ano de acompanhamento, não foram observados casos de paraplegia, paraparesia ou AVC. Nenhum evento adverso relacionado ao dispositivo foi encontrado. Em relação aos eventos adversos relacionados ao procedimento, 16% dos pacientes apresentaram eventos isquêmicos no membro superior esquerdo por obstrução intencional da artéria subclávia esquerda (8%) ou lesões relacionadas ao sítio de punção (8%). Foi necessária a revascularização da artéria subclávia esquerda em 8% dos pacientes. A mortalidade no período estudado foi de 12%, sendo que 8% dos pacientes morreram nos primeiros 30 dias de seguimento.

García Reyes et al.^15^ realizaram um estudo para investigar os resultados em longo prazo dos pacientes vítimas de trauma contuso de aorta torácica tratados pela técnica endovascular. A média de seguimento dos participantes foi de 98 meses e a principal complicação observada foi trombo intrastent (20%). Apenas 9% apresentaram necessidade de reintervenção, sendo que, em metade, foi realizada revascularização da artéria subclávia esquerda e, na outra metade, realizou-se uma reabordagem na aorta devido à presença de *endoleak* ou trombo oclusivo na endoprótese. Todas as reintervenções apresentaram sucesso e nenhuma complicação adicional foi registrada. Nenhum paciente apresentou paraplegia ou lesões neurológicas e nenhum caso de morte foi observado no período perioperatório ou durante o seguimento.

No presente estudo, apenas dois pacientes apresentaram complicações relacionadas ao procedimento. Nesses casos, a conduta expectante foi adotada e ambos evoluíram sem novas intercorrências. No seguimento ambulatorial, o controle foi realizado com angiotomografia, entre 30 a 90 dias do procedimento e em 6 meses. Após esse período, é realizada nova angiotomografia após 36 meses (3 anos) e 60 meses (5 anos) do procedimento.

Outro aspecto importante relacionado a esse tratamento envolve o *oversizing* a ser utilizado nesses pacientes, já que na maioria dos casos os indivíduos são jovens e com aorta torácica previamente sadia. Como as endopróteses não foram originalmente fabricadas para o tratamento de lesão de aorta traumática, esses dispositivos apresentam estrutura compatível com aortas de maiores diâmetros, como na doença aneurismática. Devido a isso, um *oversizing* excessivo muitas vezes é inevitável em pacientes com lesão contusa de aorta devido à falta de endopróteses de tamanho adequado^15^. As recomendações atuais na literatura são para utilizar um *oversizing* máximo de 20%^16^. O tamanho da endoprótese e sua posição são fatores determinantes nos resultados após o tratamento. García Reyes et al.^15^ observaram que pacientes que apresentaram complicações relacionadas à endoprótese possuíam um *oversizing* maior que o grupo sem complicações (p = 0,0007)^15^.

Os materiais disponíveis atualmente, em sua maioria, não são específicos para lesões traumáticas de aorta, sendo difícil encontrar tamanhos menores e ideais para pacientes jovens^16^. Esse fato obriga muitos cirurgiões a implantarem endopróteses com *oversizing* maiores, como única alternativa viável para o tratamento endovascular. Entretanto, o *oversizing* excessivo pode estar relacionado com *endoleak* tipo 1, acotovelamento e colapso da endoprótese^17^.

Entre as endopróteses liberadas para uso, destacam-se a Valiant® (Medtronic), a C-TAG® (L. Gore & Associates, Inc.) e a TX2® (Cook Group Inc.). Entretanto, deve-se ponderar a disponibilidade desses materiais nos serviços, bem como a anatomia do paciente para escolha do material adequado. A conformabilidade da endoprótese, diminuindo o *spring-back force*, diminui também a possibilidade de *endoleaks* futuros ou colapso da endoprótese, o que pode levar a melhor indicação para esses casos^17^. Os perfis das endopróteses torácicas variam em média entre 18-24F, o que muitas vezes pode ser incompatível com o diâmetro das artérias femorais para implante das endopróteses torácicas, propiciando, dessa forma, a preferência para endopróteses *low profile*, com calibres menores.

O diâmetro da aorta proximal à lesão variou entre 19 mm e 30 mm, sendo que o maior diâmetro estava relacionado com uma paciente de 67 anos que possivelmente apresentava dissecção crônica prévia (caso 4 – Tabela 2). Com relação ao *oversizing*, variou entre 11 e 21%. O objetivo inicial era de um *oversizing* próximo a 10-15%, porém, como previamente descrito, houve variações devido à disponibilidade de material, sendo em muitos casos utilizado o menor diâmetro disponível na ocasião.

Novos estudos tentam estabelecer o melhor momento para o tratamento endovascular aos pacientes com lesão de aorta por trauma contuso. Algumas pesquisas já mostraram vantagem na abordagem tardia (após 24 horas) em relação à abordagem precoce (nas primeiras 24 horas), mesmo em pacientes com lesões associadas de grave intensidade^18^. Marcaccio et al.^18^ demonstraram em uma amostra de 507 pacientes que uma abordagem tardia se associa a menores taxas de mortalidade (5,4%) quando comparada ao tratamento precoce (11,9%). Além disso, a abordagem precoce se associou a um maior risco de mortalidade em uma análise de regressão logística multivariada (OR 2,39; IC 95% 1,01-5,67; p = 0,047).

Apesar de até o momento não existir nenhum estudo randomizado e controlado que compare o tratamento endovascular com a cirurgia aberta para pacientes vítimas de lesão de aorta por trauma contuso, houve melhora significativa nas taxas de mortalidade e morbidade à medida que a abordagem endovascular suplantou a cirurgia aberta convencional na maioria dos centros de trauma^12^^,^^18^.

## CONCLUSÃO

O método endovascular é uma alternativa viável no tratamento de lesões de aorta torácica por trauma contuso, conforme embasamento na literatura e na experiência do serviço. A aplicabilidade e a menor morbimortalidade são fatores a serem ponderados na escolha para a técnica endovascular. São necessários estudos randomizados e controlados, ou ainda seguimento a longo prazo desses pacientes, para reforçar a indicação desse método como terapia para esse tipo de lesão.
